# Psychophysiological arousal and inter‐ and intraindividual differences in risk‐sensitive decision making

**DOI:** 10.1111/psyp.12627

**Published:** 2016-02-29

**Authors:** Bettina Studer, Benjamin Scheibehenne, Luke Clark

**Affiliations:** ^1^ Behavioural and Clinical Neuroscience Institute (BCNI), Department of Psychology, University of CambridgeCambridgeUK; ^2^ Institute of Clinical Neuroscience and Medical Psychology, Medical Faculty, University of DusseldorfDusseldorfGermany; ^3^ Geneva School of Economics and ManagementUniversity of GenevaGenevaSwitzerland; ^4^ Centre for Gambling Research at UBC, Department of Psychology, University of British ColumbiaVancouverBritish ColumbiaCanada

**Keywords:** Decision making, Peripheral arousal, Risk, Personality, Bayesian

## Abstract

The current study assessed peripheral responses during decision making under explicit risk, and tested whether intraindividual variability in choice behavior can be explained by fluctuations in peripheral arousal. Electrodermal activity (EDA) and heart rate (HR) were monitored in healthy volunteers (*N* = 68) during the Roulette Betting Task. In this task, participants were presented with risky gambles to bet on, with the chances of winning varying across trials. Hierarchical Bayesian analyses demonstrated that EDA and HR acceleration responses during the decision phase were sensitive to the chances of winning. Interindividual differences in this peripheral reactivity during risky decision making were related to trait sensitivity to punishment and trait sensitivity to reward. Moreover, trial‐by‐trial variation in EDA and HR acceleration responses predicted a small portion of intraindividual variability in betting choices. Our results show that psychophysiological responses are sensitive to explicit risk and can help explain intraindividual heterogeneity in choice behavior.

Deciding between options with uncertain outcomes is a complex process, and the question of how we choose between different prospects and response options intrigues psychologists and economists alike. One core insight obtained from recent neuroscience research is that decision making comprises not only cognitive but also emotional processes (e.g., Coricelli, Dolan, & Sirigu, 2007; Damasio, 1994, 1996; Heilman, Crişan, Houser, Miclea, & Miu, 2010). For instance, when making decisions about risky gambles, participants are unduly biased by the anticipation of regret or envy (negative emotional states; Coricelli et al., 2005; Coricelli & Rustichini, 2010). Other research has shown that mood manipulations through exposure to emotional stimuli impact subsequent (unrelated) economic decisions, such as the price requested in selling transactions (Lerner, Small, & Loewenstein, 2004) and risk judgments (Johnson & Tversky, 1983).

Emotional processing can also be investigated via the measurement of peripheral responses (e.g., Bradley, Codispoti, Cuthbert, & Lang, 2001; Cuthbert, Bradley, & Lang, 1996; Lang, 1995; Lang & Davis, 2006). In the field of human decision making, this approach was pioneered by Bechara and colleagues (Bechara, Damasio, Tranel, & Damasio, 1997), who assessed electrodermal activity (EDA) during the Iowa Gambling Task (IGT; Bechara, Damasio, Damasio, & Anderson, 1994). In the IGT, players choose between four card decks associated with different win and loss contingencies. Two decks are advantageous, yielding a net profit (termed the *safe* or *good* decks); the other two decks deliver a net loss (termed the *risky* or *bad* decks). Bechara et al. (1997) found that EDA is higher before choices from risky decks than choices from safe decks (for replications, see, e.g., Bechara, Damasio, Damasio, & Lee, 1999; Crone, Somsen, Van Beek, & Van Der Molena, 2004; Tchanturia et al., 2007). The IGT tests decision making under ambiguity, and requires learning the reward contingencies of the four decks through trial and error. In the Somatic Marker Hypothesis, Damasio and colleagues proposed that peripheral signals convey the emotional value of choice options built up by past experience and thereby guide ongoing decisions (Damasio, 1994). An unresolved question is whether psychophysiological arousal is also sensitive to the riskiness of a decision in conditions that do not require learning. The small number of experiments that assessed peripheral arousal during decision making under explicit risk suggests this might indeed be the case: EDA and heart rate (HR) responses during risky choice were found to vary as a function of the probability of winning/losing (Holper, ten Brincke, Wolf, & Murphy, 2014; Studer & Clark, 2011) and the variance of outcomes (Holper, Wolf, & Tobler, 2014; but see also Minati et al., 2012).

Another intriguing finding in previous research is that, like choice behavior, peripheral reactivity during decision making varies considerably across individuals (see Carter & Pasqualini, 2004; Crone et al., 2004; Goudriaan, Oosterlaan, de Beurs, & van den Brink, 2006; Guillaume et al., 2009). The identification of the factors underlying this interindividual variance has clinical relevance to mental health problems characterized by aberrant sensitivity to rewards, punishments, and uncertainty, including addictions (Verdejo‐García, Lawrence, & Clark, 2008; Voth et al., 2014; Yen, Ko, Yen, Chen, & Chen, 2009) and anxiety disorders (Maner et al., 2007; Tang, van den Bos, Andrade, & McClure, 2012). Previous research has linked interindividual differences in risk‐taking behavior to a number of personality traits, most notably impulsivity (Bayard, Raffard, & Gely‐Nargeot, 2011; Christodoulou, Lewis, Ploubidis, & Frangou, 2006; Dir, Coskunpinar, & Cyders, 2014; Franken, van Strien, Nijs, & Muris, 2008; Sweitzer, Allen, & Kaut, 2008), reward sensitivity (Franken & Muris, 2005; Kim & Lee, 2011; Suhr & Tsanadis, 2007), and punishment sensitivity (Kim & Lee, 2011; Studer & Clark, 2011; Studer, Pedroni, & Rieskamp, 2013). Gender may also play a role: males show a small but reliable tendency to make more advantageous decisions than females on the IGT (see meta‐analysis by Cross, Copping, & Campbell, 2011), and an IGT neuroimaging study found gender differences in brain activation patterns (Bolla, Eldreth, Matochik, & Cadet, 2004). However, to date, interindividual differences in peripheral sensitivity during decision making have not been explored as a function of gender.

The current study assessed EDA and HR responses during the Roulette Betting Task (RBT; Studer, Apergis‐Schoute, Robbins, & Clark, 2012; Studer & Clark, 2011; Studer, Manes, Humphreys, Robbins, & Clark, 2015). In this task, participants are presented with risky gambles and decide how much they want to bet on them. Across trials, the explicitly stated chances of winning are varied (40%, 60%, or 80%). To further isolate the decisional processes involved in bet selection, the task compares two choice conditions. In the active‐choice trials, the participant selects the bet amount, whereas in the passive no‐choice trials, the bet size is dictated by the computer. We used hierarchical Bayesian analyses to analyze peripheral responses during the selection phase of active and passive decision making. Bayesian methods provide a number of advantages over conventional null‐hypothesis‐significance testing (NHST) with associated *p* values (Kruschke & Vanpaemel, 2015). Unlike NHST, the Bayesian approach allows estimating of the actual probability of a research hypothesis. Bayesian analyses also provide the full posterior probability distribution of the model parameters of interest, make prior assumptions explicit, make parameter correlations transparent, and provide a coherent framework to model hierarchical data structures (Kruschke, 2011; Scheibehenne & Pachur, 2015). Hierarchical Bayesian techniques further account for varying degrees of measurement error on the individual level and thereby improve estimation accuracy via a mechanism referred to as borrowing strength (Gelman & Hill, 2007).

Our analyses focused on the selection phase of the task specifically. This period allows characterization of signals related to decision making per se (including processing and comparison of the available choice options and actual selection). Whereas feedback*‐*related peripheral responses (i.e., responses to wins and losses) have been extensively characterized in prior research (see, e.g., Crone et al., 2004; Lole, Gonsalvez, Barry, & Blaszczynski, 2014; Lole, Gonsalvez, Blaszczynski, & Clarke, 2012; Stankovic, Fairchild, Aitken, & Clark, 2014; Starcke, Wolf, Markowitsch, & Brand, 2008; Wilkes, Gonsalvez, & Blaszczynski, 2010), few previous experiments have examined selection‐related peripheral signals under conditions of explicit risk. In our first analysis, we tested whether three peripheral indices (EDA, HR deceleration, and HR acceleration responses) were sensitive to the probability of winning/losing during bet selection, and whether these responses were moderated by the choice condition (active vs. passive). We predicted that peripheral responses would be more sensitive to the chances of winning during active selection than computer‐dictated bet selection.

Second, we tested whether interindividual differences in this peripheral sensitivity to the chances of winning were explained by trait impulsivity, reward sensitivity, and punishment sensitivity (measured by established personality questionnaires), and whether peripheral sensitivity during risky decision making varied as a function of gender.

Our final aim was to assess whether peripheral arousal can help understand intraindividual variability in choice behavior. When confronted with the same choice situation multiple times, a decision maker will not always select the same option (see, e.g., Camerer, 1989; Hey, 2001; Mosteller & Nogee, 1951). In some situations, varying one's behavior might be adaptive, for instance, during a chess or poker game against a skilled opponent. In tasks such as the IGT where reward contingencies are learned through trial and error, sampling and exploration of the different choice options is even necessary. However, in situations where these reward contingencies are provided explicitly, such instability in choice is harder to explain. In line with several influential decision theories postulating that emotional arousal can serve as an input signal in decision making—including the Somatic Marker Hypothesis (Damasio, 1994, 1996), Risk as Feelings (Loewenstein, Weber, Hsee, & Welch, 2001), Decision Affect Theory (Mellers, Schwartz, Ho, & Ritov, 1997; Slovic, Finucane, Peters, & MacGregor, 2007), and Affect as Information (Schwarz & Clore, 1983, 2003)—we hypothesized that fluctuations in peripheral arousal may constitute one source of this intraindividual variability in choice behavior. Thus, we tested whether trial‐by‐trial EDA and HR responses on the active‐choice trials predicted (residual) variance in individuals’ betting decisions that was not explained by the chances of winning.

## Method

### Participants

Sixty‐eight healthy university students (25 males, 43 females, *M*_age_ = 25 years, *SD*_age_ = 3.5 years) who had no history of psychiatric disorders and were fluent English speakers took part in this study. All participants were screened with the Problem Gambling Severity Index (Ferris & Wynne, 2001) to exclude volunteers with gambling problems.

### Procedure

Participants attended a single testing session lasting approximately 60 min, in which they first completed the questionnaires, followed by the setup of the psychophysiological monitoring and performance of the RBT. Participants gave written informed consent. The study was approved by the local ethics committee and conducted in accordance with the Declaration of Helsinki. Participants received a financial reimbursement that was partially determined by their final score on the RBT, to ensure that decisions on the task had direct financial relevance to the participant. Specifically, participants received £5 for certain plus a task‐dependent bonus that varied between £0 and £5.

### Questionnaire Measures

Participants were administered the BIS/BAS scale (Carver & White, 1994) and the Barratt Impulsiveness Scale (Version 11; Patton, Stanford, & Barratt, 1995). The BIS/BAS scale contains 24 items and measures trait sensitivity of two motivation systems, the behavioral activation system (BAS) and the behavioral inhibition system (BIS), which are postulated to drive affective responses to the anticipation and occurrence of rewards and punishment, respectively (cf. Gray, 1972, 1981). The Barratt Impulsiveness Scale consists of 30 items that measure three subdimensions of impulsivity: nonplanning impulsiveness, motor impulsiveness, and attentional impulsiveness. Our analyses focused on nonplanning impulsivity, defined as the tendency to make rash decisions without much forethought or planning (Patton et al., 1995), which is the most relevant subdimension for decision‐making behavior (see Christodoulou et al., 2006; Malloy‐Diniz, Fuentes, Leite, Correa, & Bechara, 2007).

### Experimental Task

**Figure 1:**
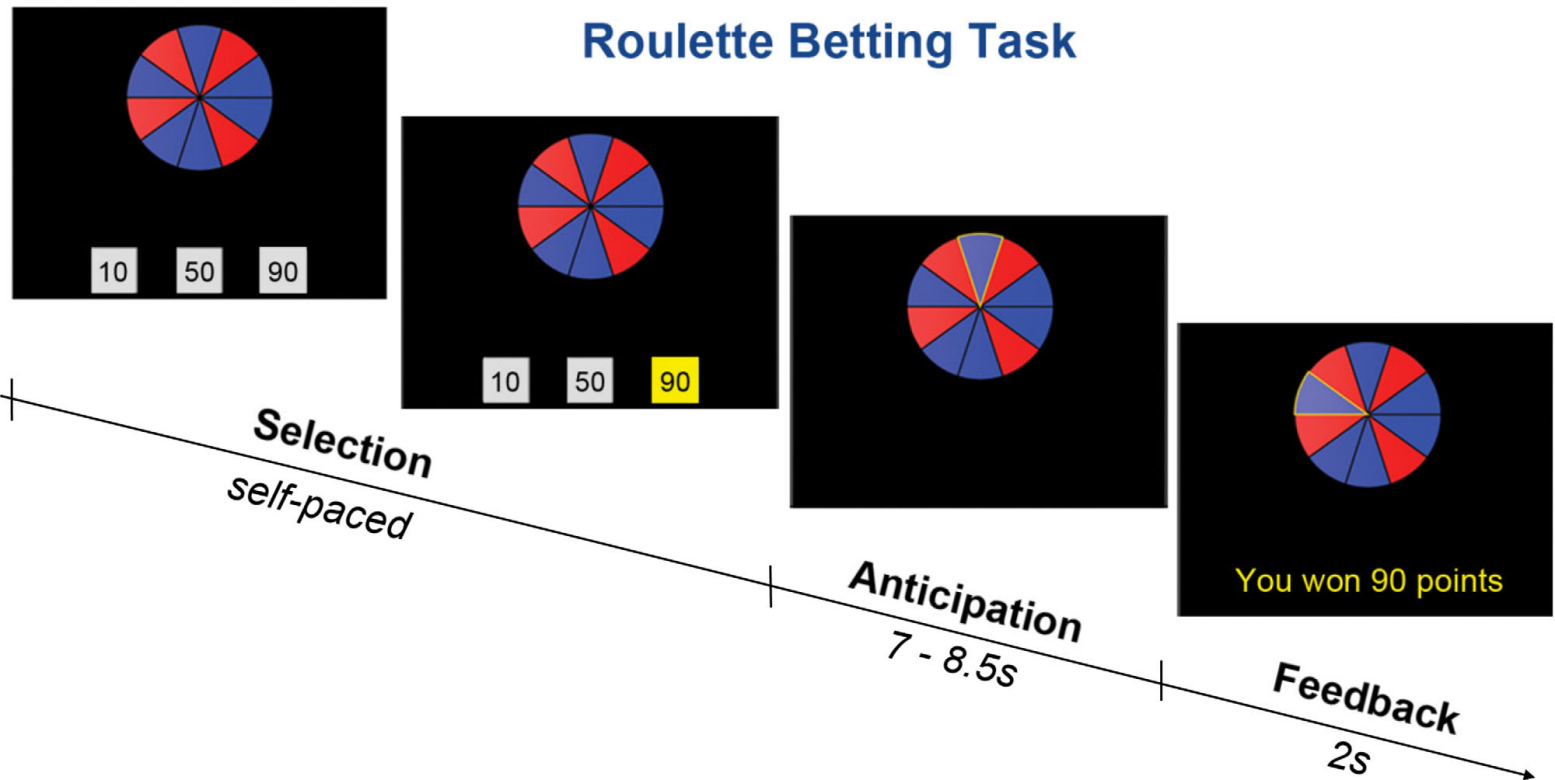
Roulette Betting Task. Each trial consisted of three phases: (1) selection, in which the participant chose one of three bet boxes, (2) anticipation, in which the wheel was spun, and (3) feedback, in which the decision outcome was presented. In active‐choice trials, participants were presented with three different bet options, while in no‐choice trials all three bet boxes contained identical amounts (not shown). Psychophysiological responses were modeled to the onset of the selection phase.

The RBT (Studer et al., 2012; Studer & Clark, 2011) was programmed in Visual Basic 2008 (Microsoft Corp., Redmond, WA) and administered on a laptop PC with keyboard control. In this task, the participant bets on roulette‐type gambles. In each trial, a wheel containing 10 blue and red segments was presented. Below the wheel, three bet boxes were visible (see Figure 1). The ratio of blue to red segments represented the chances of winning. Blue segments were designated winning segments, and red were losing segments. Across trials, two parameters were varied: the chances of winning (three levels: probability of winning versus losing = 40% vs. 60%, 60% vs. 40%, or 80% vs. 20%) and the choice condition (two levels: active‐choice [A] vs. no‐choice [N]). The task thus contained six trial types, henceforth labeled A_40%_, A_60%_, A_80%,_ N_40%_, N_60%_, N_80%_ (with the percentage number indicating the probability of winning). In the active‐choice trials, the bet boxes presented three options (10, 50, or 90 points), and the participant selected their bet amount. In the no‐choice trials, the three boxes contained identical amounts. Thus, in both conditions, the participant was required to make a (self‐paced) key press to select one of the three bet boxes, ensuring that motor requirements were matched and only the active‐choice trials required a risk‐sensitive bet decision. Once a response was made, the wheel spun for a variable duration (7–8.5 s) and then stopped on a segment. If the wheel stopped on a blue segment, the bet was won, and the outcome message “YOU WON [XX] POINTS” was presented. If the wheel stopped on red, the bet was lost, and the message “YOU LOST [XX] POINTS” appeared. At the end of each trial, a fixation cross was displayed for a variable intertrial interval (8–10 s), to allow recovery of physiological responses.

Participants completed a total of 90 trials (15 trials of each type), divided into three blocks. At the end of each block, the accumulated score was presented.

In addition to being informed about these task rules, participants were instructed to try to win as many points on the task as possible, and were told that their accumulated total score would determine a bonus payment based on a conversion chart.

### Acquisition of Peripheral Data

A BIOPAC system (MP150, recording at 1,000 samples per second; BIOPAC Systems, Inc., USA) with two amplifiers (ECG100C module and GSR100C module) was used to measure EDA and HR. Electrodes were attached prior to beginning the task, and 5 min of resting state activity were recorded to allow participants to adapt to the recording equipment and for EDA levels to stabilize (see Fowles et al., 1981). EDA was measured using two grounded Ag‐AgCl electrodes attached to the distal phalange of the index and middle fingers of the nondominant hand. Isotonic paste (BIOPAC Gel 101) was used as the electrolyte. A low‐pass filter of 1.0 Hz and a DC high‐pass filter were applied to the EDA recording, and the signal was transformed to micro‐Siemens units. HR was recorded using disposable Ag‐AgCl electrocardiogram (ECG; Vermed EL503) patches secured to the right dorsal forearm and left ankle, and high‐pass filtered at 0.05 Hz. The interbeat intervals were calculated from deviations between the R waves and transformed online to beats per minute (bpm). AcqKnowledge Software (Version: 3.9.0, BIOPAC Systems, Inc.) was used to record and event‐mark the psychophysiological data.

### Extraction of Peripheral Responses

EDA and HR responses were modeled to the presentation of the wheel, and extracted for each trial. A 1‐s window immediately preceding the wheel onset served as a trial‐by‐trial baseline. EDA responses were calculated as the maximum value in the time window 2–7 s after wheel onset minus baseline (Dawson, Schell, & Filion, 2007). HR responses to motivational stimuli are typically multiphasic, with an initial HR deceleration within 1–3 s and a subsequent rebound acceleration (see Bradley, 2000, 2009; Bradley & Lang, 2007; Graham & Clifton, 1966; Hodes, Cook, & Lang, 1985, for reviews). Therefore, for each trial, two HR measures were extracted: HR deceleration, defined as the minimum value in the time window 0–3 s after wheel onset minus baseline, and HR acceleration, defined as the maximum value in the time window 2–6 s after wheel onset minus baseline. Thus, for HR deceleration, greater negative peak values indicated larger responses, whereas for HR acceleration and EDA, greater positive peak values indicated larger responses. The extracted trial‐by‐trial EDA and HR responses were then standardized (*z*‐scored) for each participant,^1^ to enable comparisons across the peripheral indices. Four participants were excluded from EDA analysis due to technical problems during data acquisition. Five participants were excluded from HR analyses due to excessive noise in their ECG recording. Therefore, final sample size for analyses of EDA and HR responses were *n* = 64 and *n* = 63, respectively.

### Statistical Analysis

Three sets of statistical analyses were conducted on the psychophysiological responses. In a first step, we tested whether peripheral responses were sensitive to the choice condition and chances of winning, using Bayesian analyses of variance (ANOVAs) for within‐subject data, as proposed by Kruschke (2010) and Kéry (2010). This method yields posterior probability distributions for the peripheral responses in each of the six trial types (3 Chances of Winning × 2 Choice Condition). Pairwise contrasts of these distributions were then calculated to investigate effects of interest. Since we hypothesized that sensitivity to the probability of winning/losing should be greater in the active‐choice condition, our primary effect of interest was the interaction between the choice condition and the chances of winning. Within our model, this effect was tested by comparing the difference between responses in trials with a high (80%) versus with a low (40%) probability of winning across the two choice conditions (i.e., A_80%_ − A_40%_ versus N_80%_ − N_40%_). To test for gender differences, we also calculated separate Bayesian ANOVAs for male and female participants, and then compared the posterior estimated peripheral responses.

A second analysis tested whether interindividual differences in peripheral sensitivity to the chances of winning could be predicted by the trait measures. Based on the results of the first analyses, EDA and HR acceleration responses in the active‐choice trials were investigated. The reactivity measures were formalized as the difference between the standardized response in A_80%_ trials and in A_40%_ trials, with a larger difference indicating that responses increased more strongly with higher chances of winning. Bayesian regression analyses with the three personality measures (Barratt nonplanning impulsivity, BIS, and BAS) as predictors were then calculated.

The final set of analyses investigated whether intraindividual variability in betting choices could be explained by the trial‐by‐trial levels of peripheral arousal during the selection period. Specifically, a Bayesian analysis of covariance (ANCOVA) tested whether the magnitude of EDA and HR acceleration responses predicted bet sizes on a trial‐by‐trial basis, after accounting for the chances of winning and interindividual differences. In other words, this analysis assessed whether peripheral arousal could help explain residual variation in an individual's betting choices that could not be accounted for by the likelihood of winning.

Bayesian analyses yield posterior probability distributions for all free parameters of the statistical models. To obtain these distributions, we relied on Monte Carlo Markov chain (MCMC) methods implemented in the software package JAGS (Plummer, 2013) called from R (version 3.0.2, R Core Team). For each analysis, we drew a total of 100,000 representative samples from the joint posterior parameter distribution using four separate Markov chains. Inspection of the autocorrelation and the mixing of the Markov chains for the relevant model parameters indicated that the sampling procedure was efficient and that samples were drawn from the whole range of the posterior distribution. The Bayesian approach further requires specification of prior probability distributions for each model parameter. Here, we specified priors that allow for a wide range of plausible values. To ensure that our choice of priors did not anticipate or promote the final results, we conducted prior sensitivity checks (Vanpaemel, 2010): All results were qualitatively unchanged when other priors were used. Sampling efficiency for all estimated model parameters was appropriate as indicated by the Gelman‐Rubin statistic and the estimated effective sample size. Full details of the model specification, including a description of the prior distributions and the actual computer codes as well as sampling efficiency information for all model parameters, can be obtained from http://scheibehenne.de/Appendix.StuderScheibehenneClark.zip

For all analyses, we report posterior means and highest posterior density intervals (HDP_95_). The HDP_95_ indicates the interval in which 95% of the most probable values for an estimated comparison or parameter fall (sometimes also referred to as credible interval). In the first set of analyses, responses in two experimental conditions were deemed credibly different when the HPD_95_ of the difference between the two conditions did not include zero. Equivalently, predictors of interindividual and intraindividual differences were deemed credible when the HPD_95_ for the corresponding beta weight in the regression equation did not include zero. In the analyses of inter‐ and intraindividual differences, we also provide Bayesian *R* ^2^ values, calculated based on the ratio between the variance of the data and the variance of the residuals (Gelman & Pardoe, 2006).

## Results

### Behavioral Data

**Figure 2:**
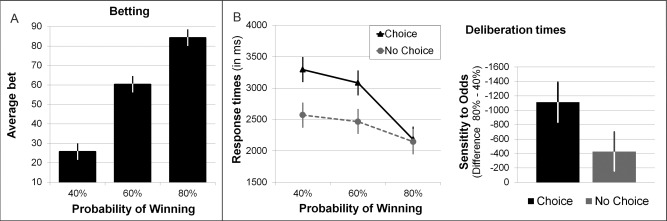
Behavioral responses. A: Participants adjusted their bets to the chances of winning in the active‐choice trials. The posterior estimated average bet size for each level of the chances of winning are displayed. B: Decision latencies decreased with increasing chances of winning, and more so in the active‐choice condition. The middle panel displays the estimated response time for each of the six different trial types. The right panel shows the sensitivity of response times to the chances of winning in the active‐choice and no‐choice conditions. Error bars represent the HPD_95_.

Prior to analyzing peripheral responses, we assessed betting behavior (in active‐choice trials) and response times. In active‐choice trials, participants adjusted their bets to the chances of winning, placing higher bets at more favorable odds (see Figure 2A). A Bayesian ANOVA for within‐subject data confirmed that bet amounts were credibly different between the three levels of odds: The estimated mean difference between A_60%_ and A_40%_ was 35 points, HPD_95_ [30 points, 39 points]; the estimated mean difference between A_80%_ and A_60%_ was 24 points, HPD_95_ [19 points, 29 points].

A 2 × 3 Bayesian ANOVA of response times revealed a credible interaction effect between the choice condition and the chances of winning (see Figure 2B): Response times decreased with increasing chances of winning in both choice conditions, and this modulation of response times by the chances of winning was credibly stronger in active‐choice compared to no‐choice trials, A_80%_ − A_40%_ = 1,100 ms, N_80%_ − N_40%_ = 424 ms, difference = 686 ms, HPD_95_ of difference [298 ms, 1,080 ms]. There was a further credible main effect of choice condition, with slower responses on active‐choice compared to no‐choice trials.

### Peripheral Sensitivity to the Chances of Winning

**Figure 3:**
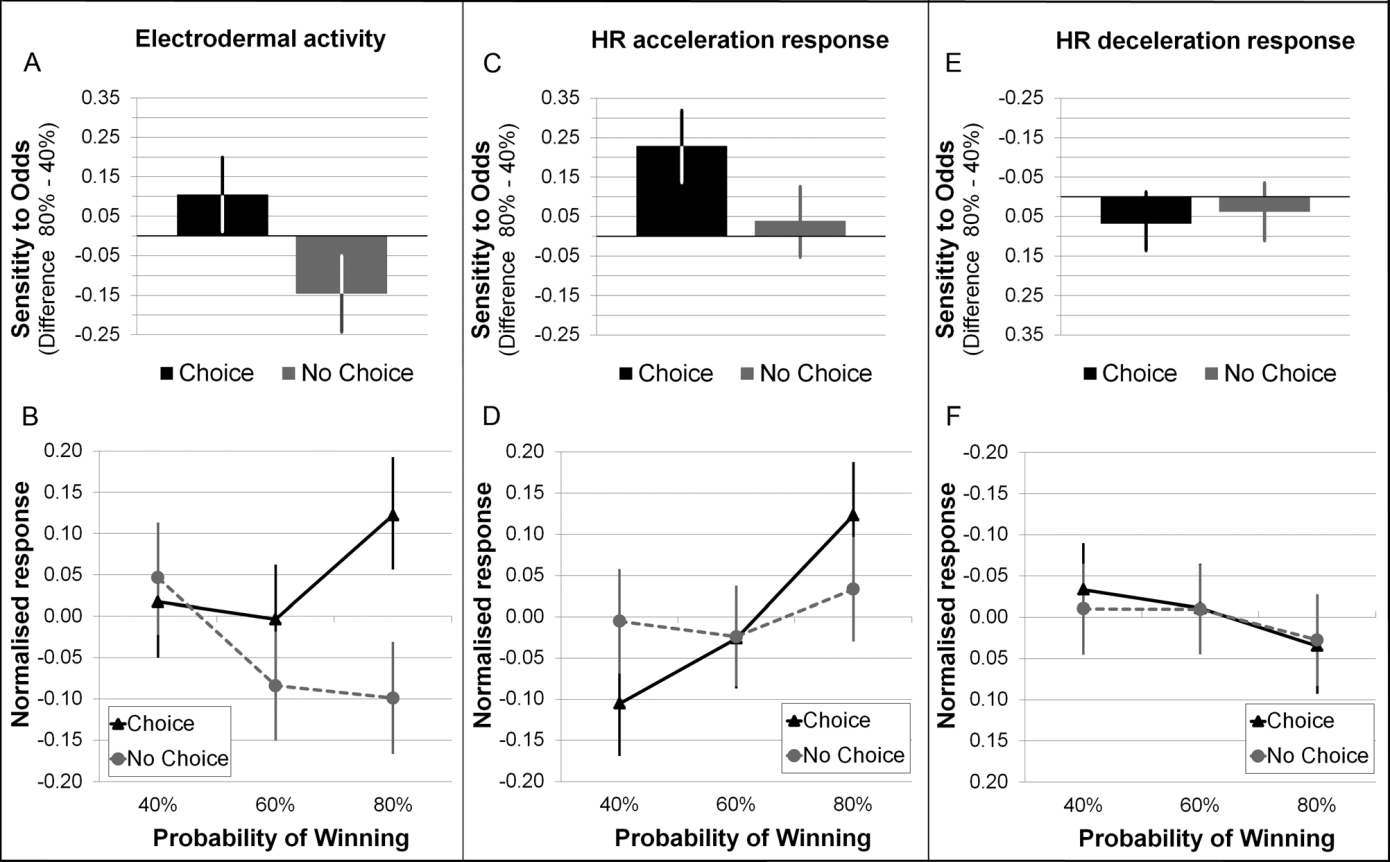
Sensitivity of peripheral responses to the chances of winning. Selection‐related EDA and HR accelerations, but not HR decelerations, reacted differentially to the chances of winning in the active‐choice versus no‐choice condition. The top panels display the sensitivity of EDA (A), HR acceleration (C), and HR deceleration (E) responses to the chances of winning in active‐choice and no‐choice trials. The bottom panels show the posterior estimated responses (standardized) for each of the six different trial types (B—EDA, D—HR acceleration responses, F—HR deceleration responses). Note that polarity of the *y* axis in the panels depicting HR decelerations (E and F) is reversed, as for HR decelerations stronger responses are represented in more negative values. Error bars represent the HPD_95._

Next, we tested whether EDA, HR decelerations, and HR accelerations during the selection phase were sensitive to the chances of winning and the choice condition. A credible interaction effect of Choice Condition × Chances of Winning was found for EDA (see Figure 3A). In active‐choice trials, EDA during the selection period increased with higher chances of winning, whereas in the no‐choice trials, EDA decreased with higher chances of winning (see Figure 3B).^2^

A similar pattern of results emerged for HR acceleration, for which a credible interaction effect was also found (see Figure 3C). HR accelerations increased with the chances of winning in active‐choice trials, but were not sensitive to the chances of winning in no‐choice trials. Figure 3D displays the posterior estimated HR acceleration response (standardized) for each trial type.

For HR deceleration response, no credible interaction effect was found (see Figure 3E). Rather, as can be seen from Figure 3F, HR deceleration responses were similar across the six trial types, indicating that HR deceleration was not sensitive to the chances of winning or choice condition.

In summary, HR acceleration and EDA during active decision making were sensitive to the chances of winning, particularly in the active‐choice condition.

### Gender, Betting Behavior, and Peripheral Responses

**Figure 4:**
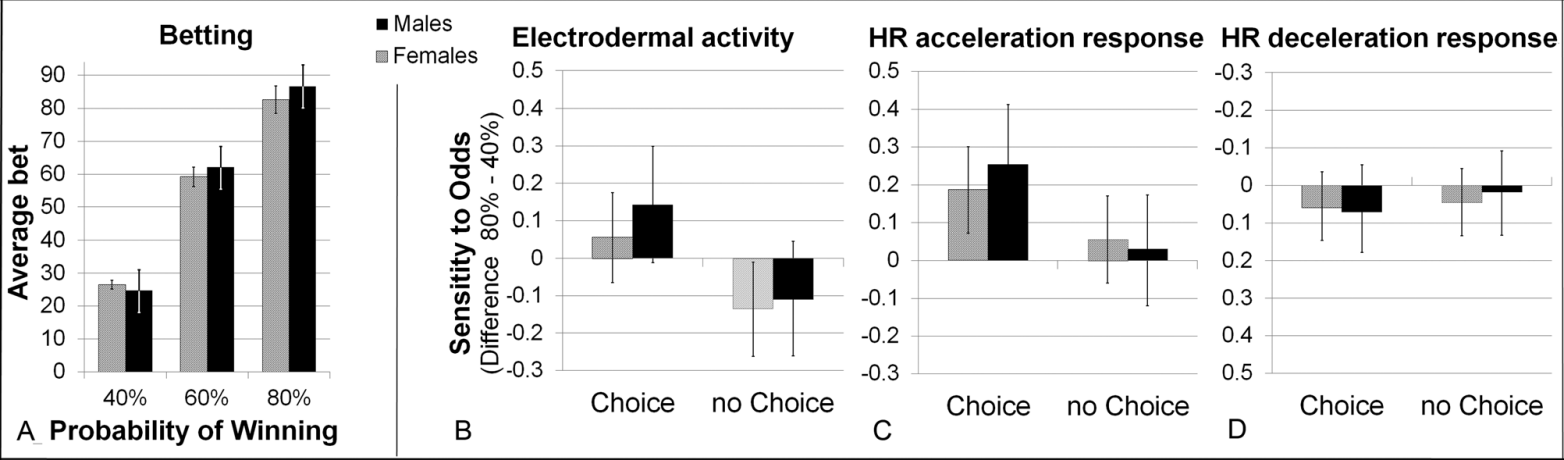
Comparison of betting and peripheral responses in male versus female participants. A: Posterior estimated bet amounts in male and female participants. B, C: Estimated sensitivity of EDA, HR acceleration, and HR deceleration responses to the chances of winning in active‐choice and no‐choice trials, estimated separately for male and female participants. Neither betting behavior nor peripheral sensitivities differed credibly between the gender subgroups. Error bars represent the HPD_95_.

Betting behavior did not differ credibly between male and female participants: a comparison of posterior bet amounts (in active‐choice trials) found no reliable gender differences (see Figure 4A).

Patterns of peripheral responding were also highly similar in female and male participants: Comparisons of the posterior mean peripheral responses in each of the six experimental conditions found no credible gender differences for any of the three peripheral indices (see online supporting information Figure S1). A direct comparison of the Choice Condition × Chances of Winning interaction effect in female and male participants found no credible difference (see Figure 4B–D).

### Personality Predictors of Psychophysiological Reactivity During Decision Making

Descriptive statistics for the three personality measures (BIS scale, BAS scale, Barratt nonplanning impulsivity subscale) are presented in Table 1. Sample means and range were comparable to those found in previous healthy samples (e.g., Carver & White, 1994; Penolazzi, Leone, & Russo, 2013; Suhr & Tsanadis, 2007), and scores were approximately normally distributed. Internal consistency was high for each scale, Cronbach's α = .75 for nonplanning impulsivity, α = .79 for BIS, α = .81 for BAS.

**Table 1:** Personality Measures

	Pooled sample (*n* = 67)			Male (*n* = 25)		Female (*n* = 42)		Bayesian comparison	
Measure	*M*	*SD*	Range	*M*	*SD*	*M*	*SD*	Difference	HPD_95_
Nonplanning impulsivity	22.0	5.1	13–35	22.0	4.1	22.1	5.6	.11	[‐2.34, 2.63]
BIS	21.1	3.6	10–27	**19.9**	**3.2**	**21.8**	**3.7**	**−2.01**	**[−3.82, −.28]**
BAS	39.5	5.3	22–50	39.6	5.0	39.4	5.5	.14	[−2.50, 2.79]

*Note*. One participant did not complete the personality questionnaires, thus the final sample size was *n* = 67. Bold indicates a credible difference between female and male subsamples.

**Figure 5:**
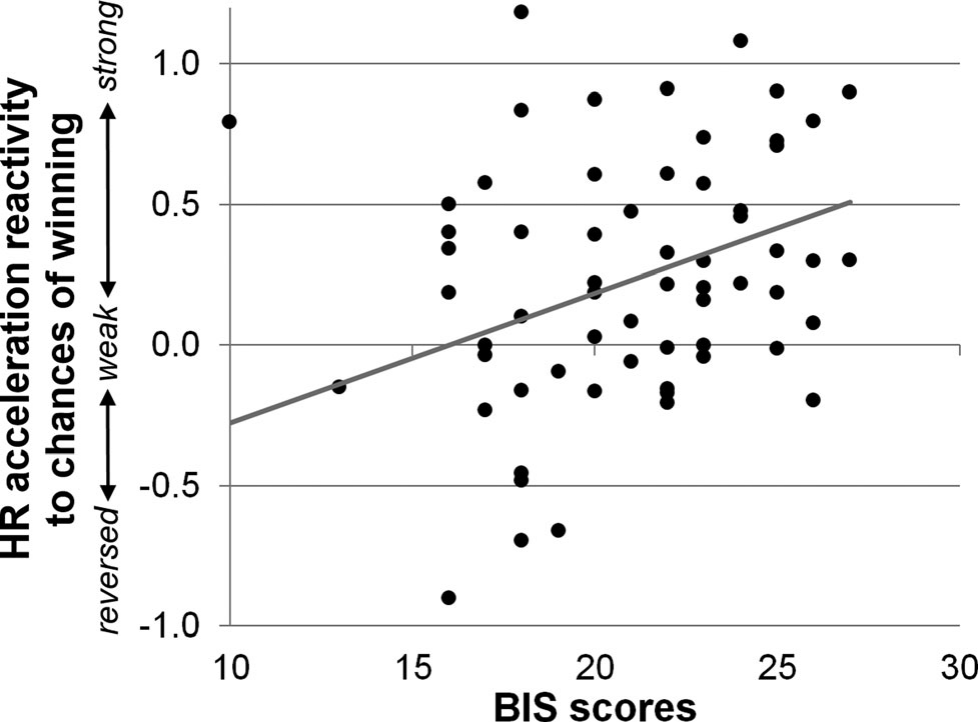
Trait sensitivity to punishment predicts peripheral sensitivity to odds. Self‐reported trait sensitivity to punishment predicted HR acceleration reactivity to the chances of winning during active bet selection. (Standardized) HR acceleration responses of participants with higher BIS scores differed more strongly between trials with high versus low chances of winning.

**Figure 6:**
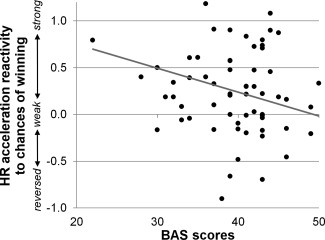
Trait sensitivity to reward negatively predicts peripheral sensitivity to odds. Self‐reported trait sensitivity to reward inversely predicted HR acceleration reactivity to the chances of winning. (Standardized) HR acceleration responses of participants with lower scores on the BAS were more strongly modulated by the chances of winning during active bet selection.

Bayesian regression analyses tested whether individual differences in the sensitivity of EDA and HR acceleration responses (quantified as the mean posterior response in A_80%_ trials − in A_40%_ trials) were predicted by these trait measures. For HR accelerations, BIS score was a credible predictor, β = .047, HPD_95_ [.012, .082], *p*(β < 0) = .005: in participants with a high BIS score, HR acceleration responses were more sensitive to the chances of winning (see Figure 5). The BAS was also a credible (inverse) predictor of HR acceleration responsiveness, β = −.03, HPD_95_ [−.05, −.001], *p*(β > 0) = .02: participants with low trait sensitivity to reward showed higher cardiac sensitivity to the chances of winning (see Figure 6). Together, these two trait predictors explained 8% of the interindividual variance in HR acceleration responsiveness, *r* ^2^ of combined regression model = .08. Nonplanning impulsivity did not credibly predict HR acceleration reactivity. For EDA, no credible trait predictors were found.

### Predicting Bet Size from Peripheral Arousal

In a final step, we assessed whether variation in HR acceleration and EDA responses could explain intraindividual differences in betting. A Bayesian ANCOVA tested whether the trial‐by‐trial magnitude of the peripheral responses predicted variation in the bet amounts, above and beyond the chances of winning. A credible—albeit small—effect of the trial‐by‐trial EDA upon (standardized) bet sizes was found, with bet amounts increasing with the magnitude of the EDA response. The mean posterior probability estimate for the corresponding beta weight was .04, HPD_95_ [.019, .066], *p*(β < 0) = .0003, *r^2^* change = .02, and the *r* ^2^ of the full model (including three predictors: the chances of winning, a random term accounting for interindividual differences, and the standardized trial‐by‐trial EDA response) was .66. The analysis of HR accelerations revealed a similar increase of bet sizes with stronger HR responses, β = .03; HPD_95_ [.0052, .057], *p*(β < 0) = .0098, *r* ^2^ change = .03, and the *r* ^2^ of the full model was .65. Thus, trial‐by‐trial HR accelerations and EDA responses were credible predictors of intraindividual betting behavior.

## Discussion

In the current study, we assessed peripheral responses during the RBT, a measure of decision making under explicit risk. Our data revealed three main findings. First, EDA and HR accelerations during the selection period were sensitive to the chances of winning, and particularly so in the active‐choice trials. Second, interindividual heterogeneity in peripheral reactivity could be linked to scores on the BIS and BAS scales: Higher trait sensitivity to punishment (BIS) and lower trait sensitivity to reward (BAS) were associated with greater cardiac sensitivity to the likelihood of winning. Third, trial‐by‐trial fluctuations in EDA and HR acceleration responses explained a (small) portion of intraindividual variability in betting.

The results of the present study provide new insights into psychophysiological responses during decision making under risk. EDA and HR accelerations during active bet selection were sensitive to the chances of winning. Previous research using the IGT showed that peripheral arousal is sensitive to the reward/punishment contingencies of decision options under ambiguity (e.g., Bechara et al., 1999, 1997; Crone et al., 2004; Tchanturia et al., 2007; Tomb, Hauser, Deldin, & Caramazza, 2002). In contrast, only a handful of prior studies investigated whether such peripheral sensitivities also manifest during decision making under explicit risk, where the reward contingencies are fully described and do not need to be learned through trial and error. Convergent with our finding that section‐related EDA and HR accelerations are sensitive to the chances of winning, a recent study by Holper, ten Brincke et al. (2014) reported that EDA during the choice phase in a sequential risk‐taking task was modulated by the probability of ruin (i.e., of losing). Two further studies tested the sensitivity of EDA to expected value and outcome variance—both composite measures that combine the probability and magnitude of potential gains and losses—under conditions of explicit risk, and found that EDA during the processing of choice options scaled positively with both of these measures (Euteneuer et al., 2009; Holper, Wolf, & Tobler, 2014). We note that in our task expected value and (in active choice trials) outcome variance correlated positively with the chances of winning, and thus it is possible that EDA and HR accelerations were driven by one of these composite scores rather than chances of winning in itself. Together, all of these findings demonstrate that psychophysiological responses are indeed sensitive to reward/punishment contingencies during decision making under explicit risk.

The aforementioned findings converge in demonstrating that peripheral arousal is sensitive to reward/punishment probability, expected value, and outcome variance during decision making under both ambiguity and under explicit risk. However, some inconsistencies exist across studies in both the directions of identified relationships and the specific peripheral components that display sensitivity. For instance, in the current study, EDA and HR accelerations during active bet selection were larger when the chances of winning were high, whereas Holper, ten Brincke et al. (2014) observed that EDA scaled positively with the probability of losing and, in our earlier study (Studer & Clark 2011), neither of these two peripheral signals was significantly associated with the chances of winning. As a second example, while IGT studies often report higher EDA during disadvantageous choices (which have low expected value; see, e.g., Bechara et al., 1999; Carter & Pasqualini, 2004; Crone et al., 2004; Tchanturia et al., 2007), Holper, Wolf, and Tobler (2014) found a positive association between selection‐related EDA and expected value under conditions of explicit risk. Clearly, peripheral responses to these decision parameters can vary as a function of the details of the decision environment. In line with this explanation, Damasio et al. argued that the same somatic marker that serves as a positive approach‐signal in one task environment can serve as a negative avoid‐signal in another task environment (Damasio, Bechara, & Damasio, 2002). Another plausible explanation is that, in addition to the main decision variables, peripheral responses are modulated by other factors and requirements of the individual tasks. For instance, Jones et al. found that the relationship between HR acceleration and deceleration responses to the presentation of risky gambles and expected value was inverted under conditions with time pressure compared to without time pressure (Jones, Minati, Harrison, Ward, & Critchley, 2011). Our own previous effect observed on HR deceleration also merits consideration. In our earlier study (Studer & Clark, 2011), HR decelerations during active bet selection were modulated by the chances of winning—a finding that was not replicated in the present study with a larger dataset. This divergence could be explained by differences in task structure, as the current study included a larger range in the chances of winning and some negative expectancy trials. Another conceivable explanation is that these discrepancies are due to the noisy nature of HR deceleration. Of the three assessed peripheral measures, HR decelerations were the least consistent across the task, indicating low reliability (see Supporting Information for details). In the absence of other data on HR decelerations in the context of decision making under risk, it is difficult to determine which of these two explanations is more likely, and further studies are needed to examine if and in which circumstances rapid cardiac deceleration responses play a role in decision making under risk.

In our personality analyses, interindividual differences in HR acceleration sensitivity to the chances of winning during active decision making were linked to trait sensitivity to reward and punishment, as measured by the BIS/BAS scale. For BIS scores, we found a positive correlation with HR acceleration sensitivity. That is to say, HR acceleration responses during bet selection differed more strongly between trials with high versus low chances of winning in individuals with higher BIS scores. This finding is consistent with previously identified relationships between the BIS and decision making: Individuals with high BIS scores take fewer risks on economic decision‐making tasks (Kim & Lee, 2011; Studer et al., 2013) and modulate their betting behavior more strongly in response to varying chances of winning/losing (Studer & Clark, 2011) than individuals with low BIS scores. Similarly, high neuroticism and high anxiety, both BIS‐related personality traits, have been associated with better IGT performance (Carter & Pasqualini, 2004; Werner, Duschek, & Schandry, 2009). Using EEG, Leue, Chavanon, Wacker, and Stemmler (2009) observed that participants with high BIS scores showed larger N2 amplitudes—an electrophysiological marker of decision conflict monitoring—during a risky choice task compared to participants with low BIS scores. Together with our current results, these findings indicate that participants with high BIS scores are more sensitive to factors such as decision risk, decision uncertainty, and the chances of winning versus losing, both in their behavior as well as in their physiological responding.

For BAS scores, a negative correlation with cardiac sensitivity was found in the current study: HR acceleration during bet selection differed more strongly between trials with high versus low chances of winning in participants with lower BAS scores. This result is consistent with previous data on the IGT, where low BAS scores have repeatedly been associated with superior task performance (Buelow & Suhr, 2013; Goudriaan et al., 2006; Penolazzi et al., 2013; Suhr & Tsanadis, 2007). Together, these findings suggest that participants with high BAS scores (i.e., high trait sensitivity to reward) are less sensitive to the likelihood of winning versus losing, both in their decision behavior as well as in their peripheral/somatic responses. Indeed, convergent with our results, two previous small sample studies found negative association between BAS‐related personality traits and peripheral sensitivity on the IGT. Mardaga and Hanseene (2012) reported that EDA during card selection on the IGT was more sensitive to the punishment frequency of the different decks in participants with low novelty seeking—a BAS‐related trait—compared to participants with high novelty seeking. Meanwhile, Goudriaan et al. (2006) found that HR decelerations immediately prior to response button presses distinguished more strongly between advantageous and disadvantageous deck choices in participants with lower BAS scores. While these studies consistently show low BAS scores to be associated with increased peripheral sensitivities during decision making, the specific peripheral marker is not fully consistent and may reflect sympathetic versus parasympathetic contributions (Bradley, 2009; Bradley & Lang, 2007; Dawson et al., 2007; Graham & Clifton, 1966).

In addition to personality, we also tested whether gender influenced betting behavior and peripheral responses during decision making. We found no support for gender as a moderator. Past work using the IGT has described some gender differences in performance (see Cross et al., 2011; van den Bos, Homberg, & de Visser, 2013), which have been corroborated in a neuroimaging study (Bolla et al., 2004). However, for decision making under explicit risk, gender effects seem less robust: In past studies, overall levels of risk taking did not differ between female and male participants, and gender only influenced choice behavior in specific situations, such as in the trial immediately following a loss (Deakin, Aitken, Robbins, & Sahakian, 2004; Lee, Chan, Leung, Fox, & Gao, 2009; Starcke et al., 2008). Taken together, these results suggest gender might play a larger role in decision making under ambiguity than under conditions of known uncertainty.

The third finding of our study was that the trial‐by‐trial magnitudes of HR accelerations and EDA during the selection phase (active‐choice trials) served as predictors of bet size, although we note that each of these measures explained only a small part of the variance in betting. In our previous study, we demonstrated that averaged selection‐related HR acceleration and EDA (uncontrolled for the chances of winning) covaried with (average) bet sizes in the active‐choice condition (Studer & Clark, 2011). The Bayesian ANCOVA used in the current study extended this earlier result to trial‐by‐trial relationships and controlled for the chances of winning; thus, it showed that peripheral arousal could help explain some residual intraindividual variation in betting decisions. The fact that people often vary their responses when presented with the same decision multiple times has been established in previous research (e.g., Camerer, 1989; Hey, 2001; Mosteller & Nogee, 1951), and this observation has led to the formulation of probabilistic models of choice (for an overview, see Blavatskyy, 2011). These models describe that an individual's choices will be subject to variability; however, they usually do not explain what causes this variability. Our study suggests fluctuations in peripheral arousal as one source of this intraindividual choice variability. Fluctuations in peripheral activity could conceivably be brought on by incidental emotions. Studies examining mood induction effects on decision making (Cryder, Lerner, Gross, & Dahl, 2008; Gray, 1999; Isen, Nygren, & Ashby, 1988; Lerner & Keltner, 2001) provide some tentative support for this proposition, and future research might test it directly by assessing whether experimental manipulation of peripheral activity—for example, pharmacologically (Sokol‐Hessner et al., 2015) or through subliminal emotional priming—systematically alter decision behavior. Another plausible explanation is that fluctuations in peripheral arousal during the selection period reflect subtle differences in the emotional valuation of the decision options at hand. For instance, the subjective valuation of a given set of choice options might be influenced by recent outcome history. Speaking somewhat against this proposition is the observation that intraindividual variability in choice behavior exists even when no outcomes are presented (Venkatraman, Payne, Bettman, Luce, & Huettel, 2009). Nonetheless, it is conceivable that such effects do occur when decision outcomes are experienced. Therefore, future studies might aim to formally test this proposition by using decision paradigms that are optimized for the testing of *n*+1 effects upon peripheral arousal.

## Conclusions

The results of the present study confirm that peripheral arousal is sensitive to the riskiness of decisions even when reward contingencies are explicitly described and no learning is required. Further, a small portion of intraindividual variation in risky choice behavior could be related to variation in peripheral arousal, suggesting that fluctuations in peripheral arousal might be one source of intraindividual differences in decision‐making behavior. Finally, we found that interindividual heterogeneity in psychophysiological sensitivity to risk taking can be partially explained by trait sensitivity to punishment and trait sensitivity to reward.

## Supporting information


**Figure S1:** Sensitivity of peripheral responses in male versus female participants.
**Appendix S1**: Internal consistency of peripheral responses
**Table S1:** Estimation of internal consistency of peripheral responses.
**Appendix S2**: Posterior model fit.
**Figure S2:** Posterior model fit of data shown in Figure 2.
**Figure S3:** Posterior model fit of data shown in Figure 3.
**Appendix S3**: Further material retrievable from authors’ website.

## Footnotes

1 Means and *SD*s of raw measures used in *z* transformation can be found at http://scheibehenne.de/Appendix.StuderScheibehenneClark.zip

2 For some participants, the distribution of the peripheral measures was slightly skewed. When rescaling the data by means of a logistic transformation, the distributions were more symmetric and the results became stronger. However, for ease of interpretation, we report all results based on the original (*z*‐scored) data.
