# Supplementary material for: Psychophysiological arousal and inter‐ and intraindividual differences in risk‐sensitive decision making
Source: Psychophysiology. 2016 Feb 29;53(6):940–50. doi: 10.1111/psyp.12627 (PMC4869679; doi:10.1111/psyp.12627)
Supplement: Supplementary file 1 — Figure S1: Sensitivity of peripheral responses in male versus female participants. Appendix S1: Internal consistency of peripheral responses Table S1: Estimation of internal consistency of peripheral responses. Appendix S2: Posterior model fit. Figure S2: Posterior model fit of data shown in Figure 2. Figure S3: Posterior model fit of data shown in Figure 3. Appendix S3: Further material retrievable from authors’ website. [file PSYP-53-940-s001.pdf]

Supplementary Material

for

# Psychophysiological Arousal and Inter- and Intra-Individual Differences in Risk-Sensitive Decision-Making

Bettina Studer

University of Cambridge and University of Dusseldorf

Benjamin Scheibehenne

University of Geneva

Luke Clark

University of Cambridge and University of British Columbia

**Table of contents**

*Supplementary Figure 1*. Estimated peripheral responses in male versus female participants. 3

Internal consistency of peripheral responses ........................................................................4

Supplementary Table 1 .........................................................................................................5

Posterior Model Fit ...............................................................................................................6

*Supplementary Figure 2A.* ...................................................................................................6

*Supplementary Figure 2B.* ...................................................................................................7

*Supplementary Figure 2C.* ...................................................................................................8

*Supplementary Figure 3A.* ...................................................................................................9

*Supplementary Figure 3B.* .................................................................................................10

Note on further material retrievable from

http://scheibehenne.de/Appendix.StuderScheibehenneClark.zip ..........................................11

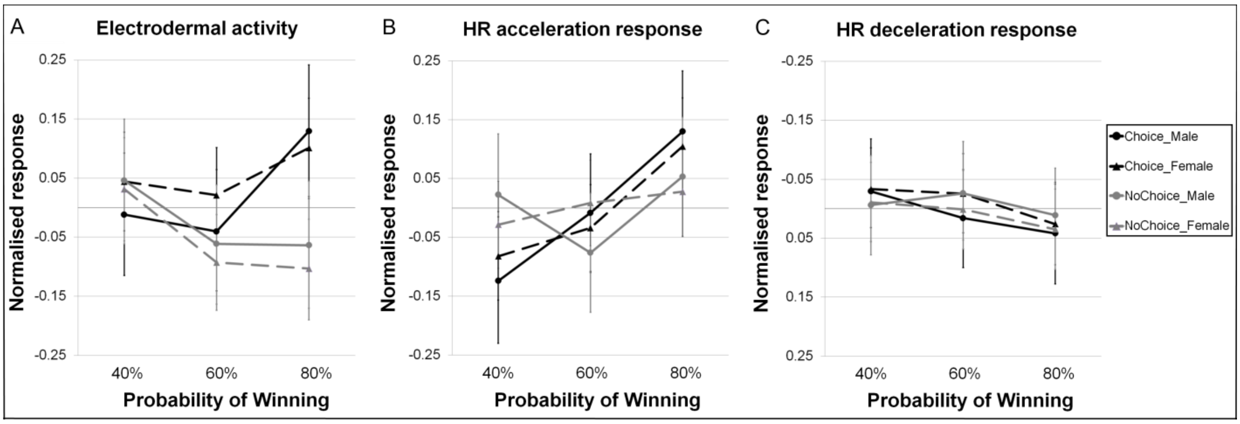


*Supplementary Figure 1*. Estimated peripheral responses in male versus female participants. Standardised, posterior EDA (A), HR acceleration (B), and HR deceleration (C) responses for each of the six different trial types, estimated separately for male and female participants. No credible gender effects were found for any measure. Error bars represent the $HPD_{95}$.

## Internal consistency of peripheral responses

In order to assess consistency of peripheral responses, the following analysis was conducted: For each of the six experimental trial types, we calculated the average response per participants twice, each time from one half of the total trials of this type (randomly chosen, mutually exclusive subsamples). Next, we calculated Pearson's correlations between these two estimates. The results are displayed in Supplementary Table 1, presented on page 5.

Three main observations were made:

First, correlation coefficients for EDA and, to a weaker degree, for HR accelerations were higher for active-choice trials than for no-choice trials. This result was expected, because the active-choice condition (in which participants actively select bet size) is predicted to lead to both larger and more reliable arousal responses than the non-choice condition.

Second, consistency estimates were lowest for HR decelerations, indicating that deceleration responses were less reliable or noisier than HR accelerations and EDA in the current context.

Third, in the case of EDA and HR acceleration, moderate to strong correlations were found in the active-choice condition. This finding indicates that internal consistency for these measures was adequate to high.

Supplementary Table 1

*Estimation of internal consistency of peripheral responses, separately for each trial type.*

| | *Chances of winning* | | |
|---|---|---|---|
| | 40% | 60% | 80% |
| **EDA** | | | |
| *Active-Choice* | .64*** | .66*** | .71*** |
| *No-Choice* | .03 | .19 | -.16 |
| **HR Acceleration** | | | |
| *Active-Choice* | .37** | .54*** | .55*** |
| *No-Choice* | .52 | .43*** | .60*** |
| **HR Deceleration** | | | |
| *Active-Choice* | .11 | .37** | .27* |
| *No-Choice* | .22 | .25* | .29* |

*Note:* Cells show Pearson's *r* for the correlation between the average responses calculated from one half of trials (of the corresponding trial type) and average response calculated from the other half of trials. Correlations that reached statistical significance are marked with stars: * = $p$ <.05, ** = $p$ <.01, *** = $p$ <.001

## Posterior Model Fit

In the following, the posterior parameter estimates are plotted against the observed data for the analyses and result presented in Figures 2 and 3 in the main manuscript. The Posterior Model Fit plots presented in the following (Supplementary Figure 2A – 3B) allow eye-balling in how far the posterior model estimates provide a reasonable summary of the empirical data.

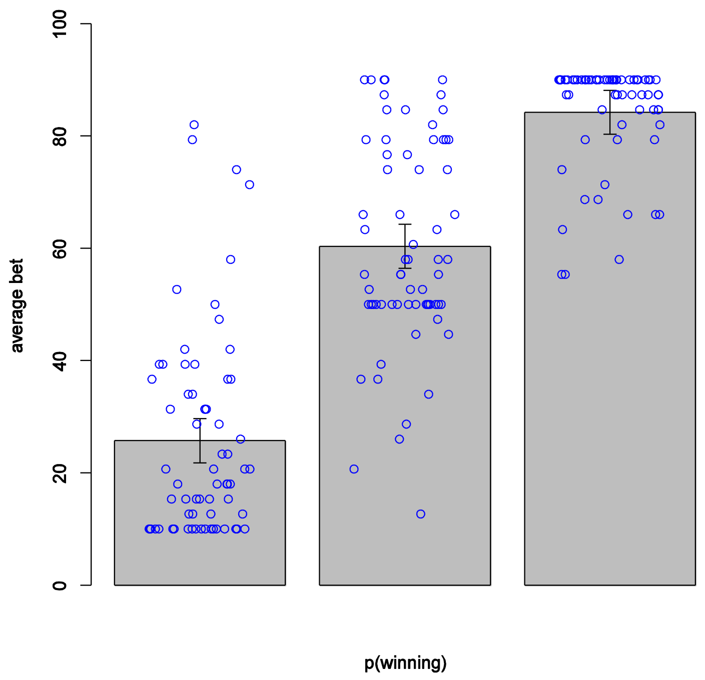


*Supplementary Figure 2A.*

Blue dots are observed data, grey bars are model estimates (means), and error bars indicate $HPD_{95}$.

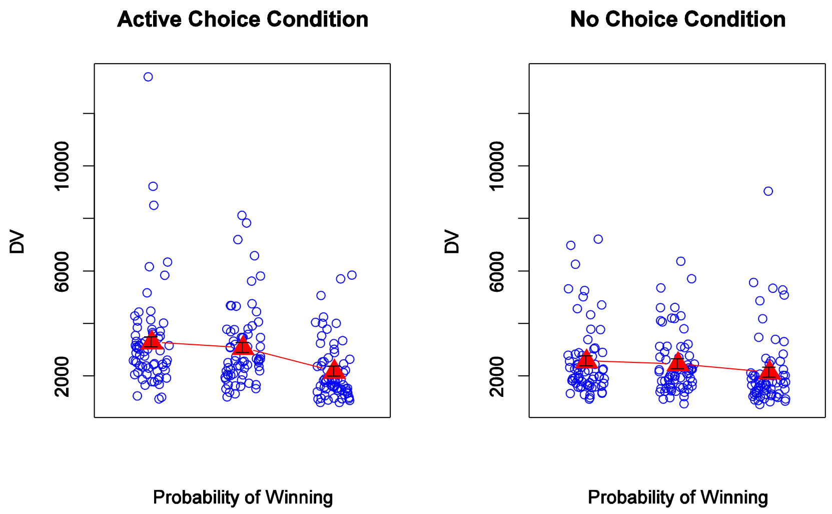


*Supplementary Figure 2B.*

Blue dots are observed data (reaction times in ms), red triangles are model estimates (means), and black error bars indicate $HPD_{95}$. Note that the response time data is positively skewed. Modelling log(response times) to avoid this skewness yields qualitatively similar results.

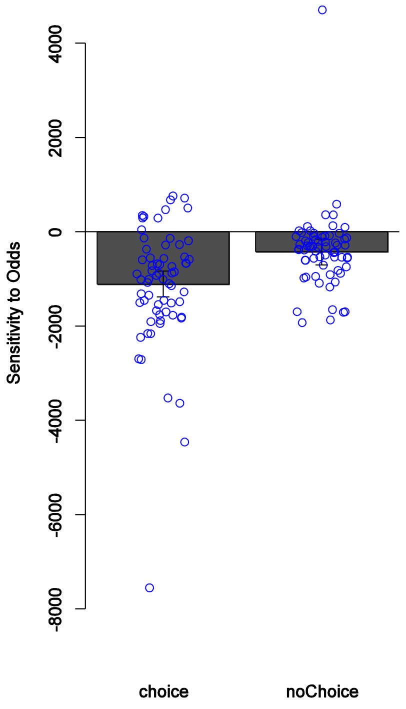


*Supplementary Figure 2C.*

Blue dots are observed data (differences in reaction times in ms), dark bars are model estimates (means), and bars indicate $HPD_{95}$. Note that the response time data is positively skewed. Modelling log(response times) to avoid this skewness yields qualitatively similar results.

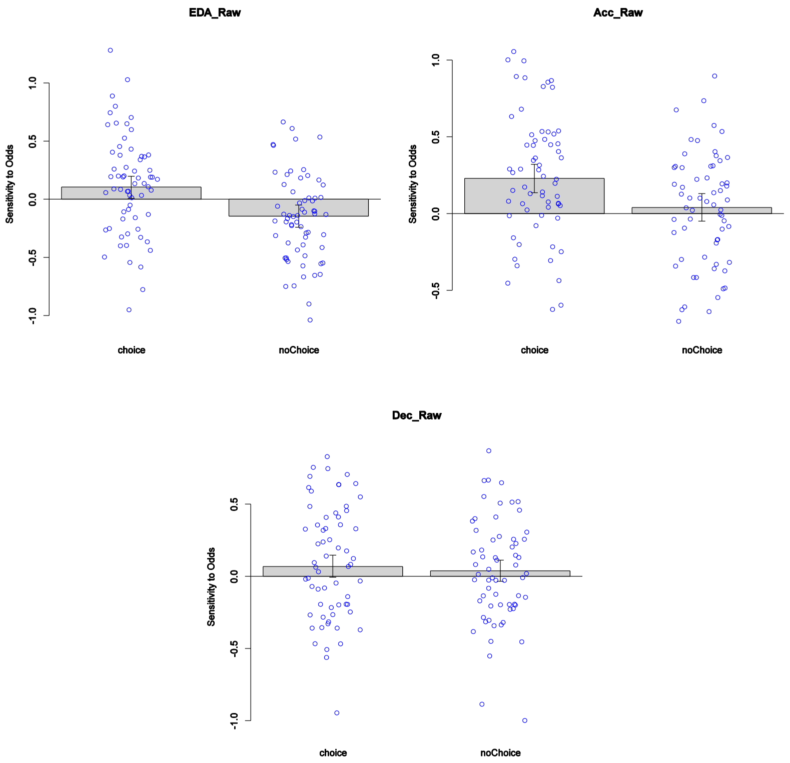


*Supplementary Figure 3A.*

Blue dots are observed data, grey bars are model estimates (means), and bars indicate $HPD_{95}$.

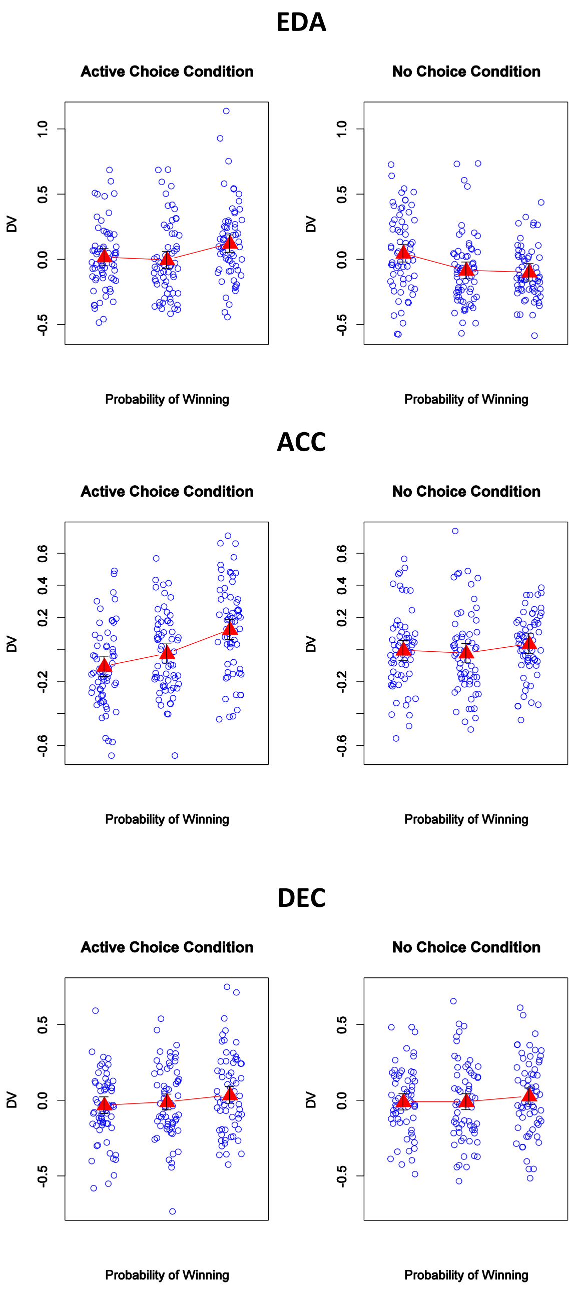


*Supplementary Figure 3B.*

Blue dots are observed data, red triangles are model estimates (means), and black error bars indicate HPD95.

## Note on further material retrievable from http://scheibehenne.de/Appendix.StuderScheibehenneClark.zip

Please note that the empirical data as well as the actual JAGS code of the Bayesian models described in the main manuscript can be downloaded from our website: http://scheibehenne.de/Appendix.StuderScheibehenneClark.zip. The corresponding R-scripts to call the Bayesian models and an overview over the sampling efficiency for all estimated models (Gelman-Rubin statistic) are also included in the aforementioned link.
